# Racial and Ethnic Differences in Insurer Classification of Nonemergent Pediatric Emergency Department Visits

**DOI:** 10.1001/jamanetworkopen.2023.11752

**Published:** 2023-05-04

**Authors:** Alexander Pomerantz, Heidi G. De Souza, Matthew Hall, Mark I. Neuman, Monika K. Goyal, Margaret E. Samuels-Kalow, Paul L. Aronson, Elizabeth R. Alpern, Harold K. Simon, Jennifer A. Hoffmann, Jordee M. Wells, Kristen H. Shanahan, Colleen K. Gutman, Alon Peltz

**Affiliations:** 1 Department of Pediatrics, Boston Children’s Hospital, Boston, Massachusetts; 2 Department of Pediatrics, Boston Medical Center, Boston, Massachusetts; 3 Children’s Hospital Association, Lenexa, Kansas; 4 Division of Emergency Medicine, Boston Children’s Hospital, Boston, Massachusetts; 5 Department of Pediatrics, Children’s National Hospital, George Washington University, Washington, DC; 6 Department of Emergency Medicine, Massachusetts General Hospital, Boston, Massachusetts; 7 Department of Pediatrics, Yale School of Medicine, New Haven, Connecticut; 8 Department of Emergency Medicine, Yale School of Medicine, New Haven, Connecticut; 9 Division of Emergency Medicine, Department of Pediatrics, Ann & Robert H. Lurie Children’s Hospital of Chicago, Northwestern University Feinberg School of Medicine, Chicago, Illinois; 10 Department of Pediatrics, Emory University School of Medicine and Children’s Healthcare of Atlanta, Atlanta, Georgia; 11 Division of Emergency Medicine, Department of Pediatrics, Nationwide Children’s Hospital, The Ohio State University College of Medicine, Columbus; 12 Department of Emergency Medicine, University of Florida, Gainesville; 13 Department of Pediatrics, University of Florida, Gainesville; 14 Department of Population Medicine, Harvard Medical School, Harvard Pilgrim Health Care Institute, Boston, Massachusetts

## Abstract

**Importance:**

Government and commercial health insurers have recently enacted policies to discourage nonemergent emergency department (ED) visits by reducing or denying claims for such visits using retrospective claims algorithms. Low-income Black and Hispanic pediatric patients often experience worse access to primary care services necessary for preventing some ED visits, raising concerns about the uneven impact of these policies.

**Objective:**

To estimate potential racial and ethnic disparities in outcomes of Medicaid policies for reducing ED professional reimbursement based on a retrospective diagnosis-based claims algorithm.

**Design, Setting, and Participants:**

This simulation study used a retrospective cohort of pediatric ED visits (aged 0-18 years) for Medicaid-insured children and adolescents appearing in the Market Scan Medicaid database between January 1, 2016, and December 31, 2019. Visits missing date of birth, race and ethnicity, professional claims data, and *Current Procedural Terminology* codes of billing level of complexity were excluded, as were visits that result in admission. Data were analyzed from October 2021 to June 2022.

**Main Outcomes and Measures:**

Proportion of ED visits algorithmically classified as nonemergent and simulated per-visit professional reimbursement after applying a current reimbursement reduction policy for potentially nonemergent ED visits. Rates were calculated overall and compared by race and ethnicity.

**Results:**

The sample included 8 471 386 unique ED visits (43.0% by patients aged 4-12 years; 39.6% Black, 7.7% Hispanic, and 48.7% White), of which 47.7% were algorithmically identified as potentially nonemergent and subject to reimbursement reduction, resulting in a 37% reduction in ED professional reimbursement across the study cohort. More visits by Black (50.3%) and Hispanic (49.0%) children were algorithmically identified as nonemergent when compared with visits by White children (45.3%; *P* < .001). Modeling the impact of the reimbursement reductions across the cohort resulted in expected per-visit reimbursement that was 6% lower for visits by Black children and 3% lower for visits by Hispanic children relative to visits by White children.

**Conclusions and Relevance:**

In this simulation study of over 8 million unique ED visits, algorithmic approaches for classifying pediatric ED visits that used diagnosis codes identified proportionately more visits by Black and Hispanic children as nonemergent. Insurers applying financial adjustments based on these algorithmic outputs risk creating uneven reimbursement policies across racial and ethnic groups.

## Key Points

**Question:**

Do insurance policies that retrospectively reduce payments for nonemergent pediatric emergency department (ED) visits by using claims algorithms introduce potential racial and ethnic differences in reimbursement?

**Findings:**

This cohort study of MarketScan Medicaid data included 8 471 386 ED visits and simulated the potential impact of 1 state Medicaid policy. Visits to the ED by Black (50%) and Hispanic (49%) children had a higher likelihood of being algorithmically identified as nonemergent and subject to professional reimbursement reductions compared with visits by White children (45%).

**Meaning:**

This study found that bias contained in the output of claims algorithms that retrospectively identify nonemergent pediatric ED visits may introduce inequity in health care financing.

## Teaser

This cohort study simulates the outcomes of an algorithm identifying nonemergent emergency department (ED) visits to model racial and ethnic differences in classification of visits by pediatric patients.

## Introduction

US health expenditures continue to increase at significant and unsustainable levels.^1^ To moderate cost growth, policy makers, clinicians, and insurers have focused on reducing wasteful spending, including avoidance of nonemergent emergency department (ED) visits. Among children, it has been estimated that up to 60% of ED visits, varying widely depending on the definition applied, may be avoided through better, more coordinated primary care.^2,3^ Recently, in many geographic locations, health insurers have enacted policies aimed at discouraging nonemergent ED visits through reductions in reimbursement for visits determined retrospectively, often by applying diagnosis-based claims algorithms, to have taken place for reasons that were nonemergent.^4,5,6^

These algorithm-based policies are expanding nationally, yet they have received intense scrutiny from advocacy groups and clinicians regarding their perceived inaccuracy and the inappropriateness of using financial reimbursement to disincentivize ED care. Particularly problematic is that these algorithms derive emergent need for ED visits from billing codes rather than the presenting symptom voiced by the patient or the necessary diagnostic process. For example, while gastroesophageal reflux is considered preventable by the algorithm, if the algorithm is unable to discern that chest pain, a potentially serious and emergent issue, was the presenting symptom, it may lead to flawed interpretations. As a result, in some settings, the policies have been delayed or reversed, but they remain active in many Medicaid programs.^7,8^

It is not well understood whether algorithmic identification of nonemergent ED visits and subsequent application of reimbursement reductions or medical necessity denials based on this output introduce racial or ethnic differences in professional reimbursement. Prior studies^9,10,11,12,13^ show that Black and Hispanic pediatric patients have different ED utilization patterns and receive different ED care compared with White patients and may have less regular access to primary and prevention services. Lower reimbursement for clinicians and/or facilities caring for these groups may translate into lower infrastructure and other investments related to the quality of care. Better understanding of the effects on equity in health care financing introduced by such policies is crucial to inform their continued use or potential expansion to new geographies or insurance groups.

Accordingly, we sought to investigate the potential equity impacts of Medicaid policies for discouraging nonemergent ED visits through algorithmic classification of diagnosis codes and application of reimbursement reductions. To do this, we modeled the potential effects of a state policy that uses a derivation of a commonly used algorithm for identifying nonemergent ED visits. We applied the algorithm to the multistate MarketScan Medicaid database (IBM Corporation) to model racial and ethnic differences in classification of nonemergent ED visits. To assess potential financial impacts, we then simulated effects of this reimbursement policy on the full multistate sample to quantify differences in reimbursement across racial and ethnic groups.

## Methods

### Study Design

We conducted a policy simulation using a retrospective cohort of pediatric ED visits (patients aged 0-18 years) appearing in the MarketScan Medicaid database between January 1, 2016, and December 31, 2019. MarketScan is a database containing patient-level demographic, enrollment, and health care claims data from 9 to 12 (depending on year) geographically dispersed and deidentified states. As we were unable to identify states in our database, we conducted a simulation exercise applying 1 state’s policy to the entire sample to simulate potential national effects on a limited scale. This study was exempted as non–human participant research by the Boston Children’s Hospital institutional review board. This study followed the Strengthening the Reporting of Observational Studies in Epidemiology (STROBE) reporting guideline for the cohort study elements of the study.

### Nonemergent ED Visits

We used the Virginia Department of Medical Assistance Services approach for identifying low-acuity and nonemergent ED visits. Hereinafter, we refer to these as nonemergent visits, since the underlying algorithm was developed to distinguish between emergent and nonemergent visits; we acknowledge that several different terminologies and conventions exist.^14^ This policy intends to enhance the efficiency of health care services by permitting managed care organizations to reduce ED clinician billed services down to the lowest billing level across all age groups for visits algorithmically determined to be nonemergent. Certain visits, such as high-severity visits and visits resulting in admissions or observation stays, are excluded.^15^ The Virginia algorithm is based on a list of nearly 800 *International Statistical Classification of Diseases, Tenth Revision, Clinical Modification* (*ICD-10-CM*) codes identified as nonemergent (eTable 1 in Supplement 1).^16,17,18^ The presence of at least 1 code in the principal position in the facility or professional claim identifies the ED visit as nonemergent. We selected this algorithm as it was among the most recent and broadscale implementations of a payment reduction policy for which there are comprehensive, publicly available technical materials that allowed us to replicate implementation to the extent possible.

### Identifying ED Visits

**Figure 1.**
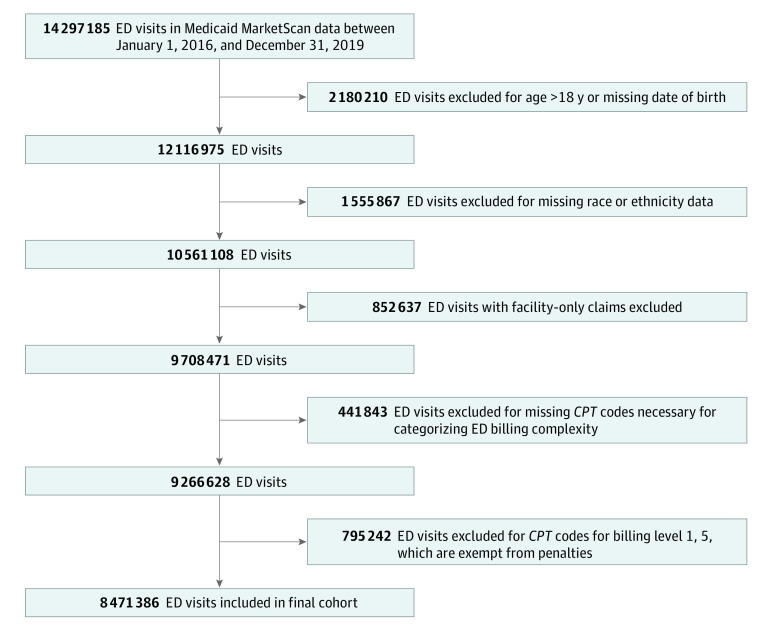
Study Cohort *CPT* indicates *Current Procedural Terminology*; ED, emergency department.

We identified children and adolescents (hereinafter referred to as children) who experienced at least 1 ED visit during the study period and had their visit categorized using *Current Procedural Terminology* (*CPT*) evaluation and management codes. *Current Procedural Terminology* codes 99281 through 99285 represent the 5 levels of billing evaluation and management for emergency services, with higher levels suggestive of more in-depth history and examination and increasing medical decision-making complexity.^19^ We limited the sample to ED visits resulting in discharge billed as *CPT* codes 99282 (level 2), 99283 (level 3), and 99284 (level 4), because these visits were potentially subject to reimbursement reduction to *CPT* code 99281 (level 1) rates under the Virginia policy. We excluded *CPT* code 99285 (level 5) visits because they were not subject to the reimbursement reduction. We excluded visits without 1 of these *CPT* codes (8.6%) and those with missing race or ethnicity information (12.8%) (Figure 1). We characterized the clinical diagnosis for each ED visit using *ICD-10-CM* billing diagnosis codes. We permitted multiple ED visits for each child.

### Main Outcome Measures

The main outcome measure was the proportion of ED visits algorithmically identified as nonemergent. We compared outcomes by age, sex, and 3 mutually exclusive race and ethnicity groups (Black, Hispanic, and White).^20,21^ The group designated as other (which included all other racial and ethnic groups outside of Black, Hispanic, and White) was removed from the simulation sample as the total enrollment (<5% of the cohort) was too small to permit stable and reliable results. Race and ethnicity were obtained through enrollment records submitted to the database developer by each participating Medicaid agency.^22^

### Statistical Analysis

We used χ^2^ tests to assess the association between each of the demographic variables and the proportion of algorithmically identified ED visits. To model financial impacts overall and sort by patient race and ethnicity, we first standardized reimbursement for the entire cohort to generate a mean reimbursement for each billing level for the overall cohort (level 2 = $28.56; level 3 = $48.39; level 4 = $83.59). Next, we applied the Virginia policy to each individual-level visit in the full cohort, reducing the actual reimbursement for those identified as nonemergent to the standardized cohort mean reimbursement for level 1 visits ($14.03), and calculating expected, population-level, per ED visit reimbursement overall and for each racial and ethnic group.^23^ This approach accounts for baseline differences in billing levels across racial and ethnic groups. All statistical analyses were conducted using SAS, version 9.4 (SAS Institute, Inc). Two-sided *P* < .05 indicated statistical significance.

## Results

The sample included 8 471 386 unique ED visits (2 747 548 [32.4%] for children aged 0-3 years; 3 642 723 [43.0%], aged 4-12 years; and 2 081 115 [24.6%], aged 13-18 years). White children (4 124 397 [48.7%]) and Black children (3 349 621 [39.5%]) had the most visits, while Hispanic children had 654 303 (7.7% of all) ED visits. Most visits were originally billed as level 3 (4 900 897 [57.9%]) and level 4 (2 896 999 [34.2%]) (Table 1). The original billing level varied by race and ethnicity with visits for Black children more often billed at level 3 (1 972 587 [58.9% of all visits]) compared with White (2 359 054 [57.2% of all visits]) and Hispanic (366 838 [56.1% of all visits]) children (*P* < .001) (Table 2).

**Table 1.** Demographic and Clinical Characteristics of ED Visits by Children

Visit characteristic	Data^a^
Race and ethnicity	
Black	3 349 621 (39.5)
Hispanic	654 303 (7.7)
White	4 124 397 (48.7)
Other^b^	343 065 (4.0)
Patient age, y	
0-3	2 747 548 (32.4)
4-12	3 642 723 (43.0)
13-18	2 081 115 (24.6)
Billing level^c^	
2	673 490 (8.0)
3	4 900 897 (57.9)
4	2 896 999 (34.2)
Professional claim reimbursement by billing level, $^c^	
2	19 234 874
3	237 154 406
4	242 160 146

Abbreviation: ED, emergency department.

^a^ Unless otherwise indicated, data are expressed as No. (%) of visits.

^b^ Includes all racial and ethnic groups with less than 5% of the total enrollment.

^c^ We analyzed only visits classified as level 2, 3, or 4. Certain ED visits are exempt from the algorithm’s reimbursement reduction, including those initially billed as level 5 (highest severity) or level 1 (as these cannot be penalized any further) and those ED visits resulting in hospital inpatient or observation status admission.

**Table 2.** Differences in Total Numbers of ED Visits by Original Billing Level and Race, Ethnicity, and Age

Characteristic	Race and ethnicity			
	Black	Hispanic	White	Other^a^
Total visits, No.	3 349 621	654 303	4 124 397	343 065
Totals visits by age group, No. (%)^b^				
0-3 y	1 145 569 (34.2)	236 187 (36.1)	1 225 673 (29.7)	140 119 (40.8)
4-12 y	1 425 360 (42.6)	287 707 (44.0)	1 789 920 (43.4)	139 736 (40.7)
13-18 y	778 692 (23.2)	130 409 (19.9)	1 108 804 (26.9)	63 210 (18.4)
No. of visits by billing level, No. (%)				
2	254 228 (7.6)	50 243 (7.7)	343 060 (8.3)	25 959 (7.6)
3	1 972 587 (58.9)	366 838 (56.1)	2 359 504 (57.2)	201 968 (58.9)
4	1 122 806 (33.5)	237 222 (36.3)	1 421 833 (34.5)	115 138 (33.6)

Abbreviation: ED, emergency department.

^a^ Includes all racial and ethnic groups with less than 5% of the total enrollment.

^b^ All comparisons across race and ethnicity groups significant at *P* < .001.

### Algorithm-Identified Nonemergent ED Visits

The algorithm identified 4 037 969 visits (47.7%) as nonemergent. A lower percentage of visits for White children (1 868 627 [45.3%]) than percentages of visits for Black (1 685 519 [50.3%]) and Hispanic (320 283 [49.0%]) children (*P* < .001) were identified as nonemergent. Visits for younger children and infants (3 144 433 [49.2%]) were more likely to be identified as nonemergent relative to visits for young adults (893 536 [42.9%]; *P* < .001) (Table 3).

**Table 3.** Algorithmically Identified Nonemergent ED Visit Characteristics

Visit characteristic	Data^a^
Race and ethnicity^b^	
Black	1 685 519 (50.3)
Hispanic	320 283 (49.0)
Other	163 540 (47.7)
White	1 868 627 (45.3)
Age, y^c^	
0-3	1 349 108 (49.1)
4-12	1 795 325 (49.3)
13-18	893 536 (42.9)
Billing level^d^	
2	271 156 (40.3)
3	2 351 447 (48.0)
4	1 415 366 (48.9)
Professional claim reimbursement reduction by billing level, $	
2	3 939 897
3	80 795 719
4	98 452 859

Abbreviation: ED, emergency department.

^a^ Percentages are calculated relative to total ED visits in each group.

^b^ All comparisons across race and ethnicity groups were significant at *P* < .001.

^c^ All comparisons across age groups were significant at *P* < .001.

^d^ All comparisons across billing level groups were significant at *P* < .001.

### Reasons for Nonemergent ED Visits

Disagreement appeared between clinician determination of severity and algorithm determination. Visits originally billed by clinicians at the lower billing level (level 2) were the least likely to be identified by the algorithm as nonemergent (40.3%). Visits billed by clinicians at higher severity levels were identified as nonemergent visits at higher rates (level 3: 48.0%; level 4: 48.9%; *P* < .001).

The most common diagnoses for algorithmically identified nonemergent ED visits were upper respiratory tract infection, cough, pharyngitis, abdominal pain, and streptococcal pharyngitis, collectively accounting for nearly one-third of all nonemergent visits. Other common diagnoses included vomiting, headache, nausea, otitis media, and rash (eTable 2 in Supplement 1).

### Reimbursement Reduction

**Figure 2.**
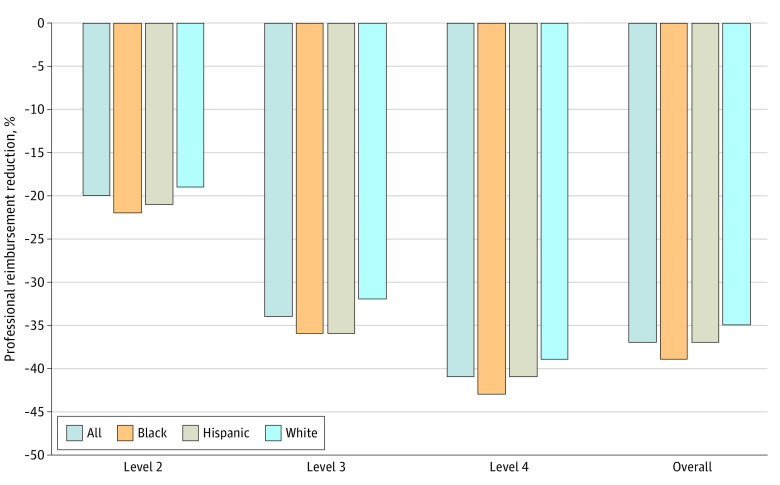
Reduction in Emergency Department Professional Reimbursement After Applying Simulated Algorithm-Derived Payment Reduction by Race and Ethnicity

The overall reimbursement for ED visits during the study period was $498.5 million. Application of the algorithm’s reimbursement reduction to the study sample would reduce professional reimbursement by $183.2 million (37% reduction). While visits for all groups were reimbursed at lower rates following application of the algorithm’s reimbursement reduction, the relative decrease was greater for ED visits for Black children (39% reduction) and Hispanic children (37% reduction) than for White children (35% reduction) (Figure 2).

Without application of the reimbursement reduction for nonemergent visits, the mean reimbursement was $58.90 per ED visit. When factoring in the effects of the reimbursement reduction, the expected mean reimbursement per ED visit across the population decreased to $37.23 with emergence of racial differences (White, $38.25; Black, $35.96; Hispanic, $37.23). This translates into 6% lower expected reimbursement per ED visit for Black children and 3% lower expected reimbursement per ED visit for Hispanic children compared with White children. When applied to the total number of ED visits across the entire sample, this resulted in $7.7 million dollars in lower relative reimbursement for Black children and $0.7 million dollars in lower relative reimbursement for Hispanic children (eTable 3 in Supplement 1).

## Discussion

In a multistate sample of more than 8.4 million ED visits by Medicaid-insured children, we found evidence of potential racial and ethnic differences in algorithmic identification of nonemergent ED visits when using billing codes. Black and Hispanic children were significantly more likely to have their ED visits classified as nonemergent. Insurers using these algorithms for reducing reimbursement for certain ED visits may inadvertently contribute to relatively lower payment for ED clinicians caring for Black and Hispanic children.

Our findings have implications for patients, providers, and policy makers on multiple levels. When clinicians and facilities receive lower reimbursement, such as what appears to occur with application of this particular algorithm, it reduces these systems’ ability to make infrastructure investments to support access to high-quality services.^24^ While we found that the relative reimbursement difference across groups was relatively small, the effects may be compounded because the clinicians and facilities serving higher numbers of Medicaid enrollees are further underfunded through these policies and other reimbursement policies. Additionally, when families risk additional out-of-pocket costs for ED visits, as seen in similar algorithms used by private insurers, they may delay or forego needed emergency care. Prior studies have found that low-income Black and Hispanic children enrolled in Medicaid use the ED at higher rates than other groups.^25,26^ Many of these differences are believed to be due to worse access to care and higher incidence of unmet social needs due to the impact of historical and contemporary discrimination. For example, transportation barriers and inadequate paid sick leave for parents create impediments for families to access timely preventive services for sick children.^27,28,29^ Without accounting for structural differences in access to the high-quality primary and prevention services needed to prevent ED visits, these algorithms, and the subsequent application of reimbursement adjustments by insurers, may risk impacting fairness in health care financing.

Appropriate interpretation of these results needs to carefully consider the theoretical nature of the study. As we did not have access to timely preimplementation and postimplementation information for Virginia (or any other state using a similar policy), this study should not be interpreted as an evaluation of current policy. Instead, we simulated potential effects in a representative state to illuminate the potential for hidden bias that might be contained in application of claims-based algorithms for determining ED reimbursement. Importantly, our study does not confirm or refute the actual presence of such bias in current use. Further, the study is able to draw inference about only the impact of algorithmic application and not potential practice-pattern variation in clinical billing across racial groups, which may serve as another potential separate mechanism for hidden bias. Finally, we standardized the simulation to a single fee schedule to isolate the effects of the policy, since states often have unique reimbursement rates. Variation in the fee schedule negotiated by certain hospitals, and differences in use of those hospitals across groups, may also lead to introduction of variation in reimbursement across groups that would not be captured in our model.

Our results also reveal that a high percentage (47.7%) of ED visits were identified by this algorithm as nonemergent visits. Previous studies have varied widely regarding both rates and operational definitions for nonemergent ED visits. One prior study by Fong^3^ described similarly high levels of nonemergent classification of ED visits in children, ranging between 51% and 56% of all ED visits. Using this same database, Samuels-Kalow et al^2^ estimated that between 1.3% and 5.7% of pediatric ED visits qualified as “low resource intensity.” Using a nationally representative survey, Raven et al^30^ found 5.5% of visits were “primary care treatable.” One recent adult study^31^ examining the impacts of a private health insurance algorithm where the financial effect was claim denial found up to 40% of the visits identified by the algorithm as nonemergent were likely clinically appropriate. The algorithm applied in the present study appears to be an adaptation of an algorithm initially developed by researchers at New York University to classify categories of ED utilization.^14^ As noted by the original developers, it was not intended as “a mechanism to determine whether ED use in a specific case is ‘appropriate’ (eg, for reimbursement purposes),” which may contribute to the very high rates of ED visits identified as nonemergent.^14^ We find evidence that this algorithm, or similar versions, were used in numerous states, for a variety of purposes ranging from program evaluation to efficiency adjustments for primary care and ED facility reimbursement rates.^32,33,34,35,36,37^ While diagnosis-based claims algorithms for classifying ED visits can be efficient tools for monitoring utilization patterns, their lack of universally accepted conventions, discordant results across administrative and clinical definitions, and potential racial and ethnic bias should serve to raise concern regarding their use in informing reimbursement policy for children.^38^

### Limitations

There are several important limitations of this study to consider. First, absent claims data specific to Virginia, our study is bound by using a separate multistate sample to model 1 state-level policy in a set of patients not exposed to the actual policy. Thus, our results are best considered a representative estimate of what might occur if this policy were rolled out in broader geographies but, importantly, should not be interpreted as a direct evaluation of the Virginia policy, which was only used as a model. While the algorithm is used to guide reimbursement in some other states, it is important to note that each state appears to use the algorithmic output in a different way. While it is plausible to assume similar bias would manifest in these states, the findings may not generalize, and they highlight the importance of preimplementation policy evaluation for potential bias. While we applied the algorithm to the extent possible using publicly available source documentation, real-world application may differ based on technical details not included in these materials. Further, we focused only on professional fee reimbursement and did not include facility charges, which are often higher than the professional fee, depending on the nature of the encounter and services provided. Professional reimbursements are more often uniform relative to the state Medicaid fee schedule as compared with facility fees, which have more complex methods and variability in negotiated rates with payors. Thus, it is possible that our results underestimate the financial effects of these policies on facilities, particularly those with employed ED clinicians. We were unable to identify clinicians or hospitals in this database and are unable to account for potential differences in professional reimbursement at specific institutions. Finally, our study relies on the completeness and accuracy of demographic data collected by state Medicaid agencies directly from enrollees. States do not require that enrollees specify their race and ethnicity but some offer incentives such as expedited processing. While states can offer many demographic categories, they are required by federal standards to classify data into 5 races and 2 ethnicities.^39^ Given that applicants self-select their choices, the likelihood of misclassification is theoretically low. One study from Minnesota matched telephone surveys with Medicaid administrative data and found 94% concordance for race and ethnicity.^40^

## Conclusions

In this cohort study of a simulation exercise including over 8 million Medicaid pediatric ED visits, we found evidence that algorithmic approaches for classifying pediatric ED care identified proportionately more visits by Black and Hispanic children as nonemergent. Insurers applying financial adjustments based on these biased outputs may risk creating differences in clinician reimbursement based on the racial and ethnic composition of the patient population.

## Notes

Supplement 1. **eTable 1.** *ICD-10* Codes Identified as Nonemergent in the Study Algorithm **eTable 2.** Common Reasons for ED Visits Algorithmically Identified as Nonemergent **eTable 3.** Algorithmically Identified Nonemergent ED Visits—Reimbursement Simulation

Supplement 2.
Data Sharing Statement
