# Supplementary material for: Racial and Ethnic Differences in Insurer Classification of Nonemergent Pediatric Emergency Department Visits
Source: JAMA Netw Open. 2023 May 4;6(5):e2311752. doi: 10.1001/jamanetworkopen.2023.11752 (PMC10160869; doi:10.1001/jamanetworkopen.2023.11752)
Supplement: Supplement 1. — eTable 1. ICD-10 Codes Identified as Nonemergent in the Study Algorithm eTable 2. Common Reasons for ED Visits Algorithmically Identified as Nonemergent eTable 3. Algorithmically Identified Nonemergent ED Visits—Reimbursement Simulation [file jamanetwopen-e2311752-s001.pdf]

Supplementary Online Content

Pomerantz A, De Souza HG, Hall M, et al. Racial and ethnic differences in insurer classification of nonemergent pediatric emergency department visits. *JAMA Netw Open*. 2023;6(5):e2311752. doi:10.1001/jamanetworkopen.2023.11752

eTable 1. *ICD-10* Codes Identified as Nonemergent in the Study Algorithm

eTable 2. Common Reasons for ED Visits Algorithmically Identified as Nonemergent

eTable 3. Algorithmically Identified Nonemergent ED Visits—Reimbursement Simulation

This supplementary material has been provided by the authors to give readers additional information about their work.

© 2023 Pomerantz A et al. *JAMA Network Open*.

eTable 1. *ICD-10* Codes Identified as Nonemergent in the Study Algorithm

ICD-10 Codes	ICD-10 Description
A09.	Infectious gastroenteritis and colitis, unspecified
J02.0	Streptococcal pharyngitis
J03.00	Acute streptococcal tonsillitis, unspecified
J03.01	Acute recurrent streptococcal tonsillitis
B01.9	Varicella without complication
B02.9	Zoster without complications
B00.2	Herpesviral gingivostomatitis and pharyngotonsillitis
B00.9	Herpesviral infection, unspecified
B09.	Unspecified viral infection characterized by skin and mucous membrane lesions
B08.5	Enteroviral vesicular pharyngitis
B08.4	Enteroviral vesicular stomatitis with exanthem
B27.80	Other infectious mononucleosis without complication
B27.81	Other infectious mononucleosis with polyneuropathy
B27.89	Other infectious mononucleosis with other complication
B27.90	Infectious mononucleosis, unspecified without complication
B27.91	Infectious mononucleosis, unspecified with polyneuropathy
B27.99	Infectious mononucleosis, unspecified with other complication
B07.9	Viral wart, unspecified
B07.0	Plantar wart
B97.11	Coxsackievirus as the cause of diseases classified elsewhere
B97.10	Unspecified enterovirus as the cause of diseases classified elsewhere
B97.89	Other viral agents as the cause of diseases classified elsewhere
A54.00	Gonococcal infection of lower genitourinary tract, unspecified
A54.02	Gonococcal vulvovaginitis, unspecified
A54.09	Other gonococcal infection of lower genitourinary tract
A54.1	Gonococcal infection of lower genitourinary tract with periurethral and accessory gland abscess
A64.	Unspecified sexually transmitted disease

© 2023 Pomerantz A et al. *JAMA Network Open*.

B35.0	Tinea barbae and tinea capitis
B35.4	Tinea corporis
B35.5	Tinea imbricata
B37.0	Candidal stomatitis
B37.83	Candidal cheilitis
B37.3	Candidiasis of vulva and vagina
B37.9	Candidiasis, unspecified
A59.01	Trichomonal vulvovaginitis
B86.	Scabies
E11.9	Type 2 diabetes mellitus without complications
E13.9	Other specified diabetes mellitus without complications
E10.9	Type 1 diabetes mellitus without complications
E11.65	Type 2 diabetes mellitus with hyperglycemia
E10.65	Type 1 diabetes mellitus with hyperglycemia
E11.69	Type 2 diabetes mellitus with other specified complication
E13.10	Other specified diabetes mellitus with ketoacidosis without coma
E10.10	Type 1 diabetes mellitus with ketoacidosis without coma
E10.69	Type 1 diabetes mellitus with other specified complication
E11.620	Type 2 diabetes mellitus with diabetic dermatitis
E11.621	Type 2 diabetes mellitus with foot ulcer
E11.622	Type 2 diabetes mellitus with other skin ulcer
E11.628	Type 2 diabetes mellitus with other skin complications
E11.638	Type 2 diabetes mellitus with other oral complications
E11.649	Type 2 diabetes mellitus with hypoglycemia without coma
E13.620	Other specified diabetes mellitus with diabetic dermatitis
E13.621	Other specified diabetes mellitus with foot ulcer
E13.622	Other specified diabetes mellitus with other skin ulcer
E13.628	Other specified diabetes mellitus with other skin complications
E13.638	Other specified diabetes mellitus with other oral complications
E13.649	Other specified diabetes mellitus with hypoglycemia without coma

© 2023 Pomerantz A et al. *JAMA Network Open*.

E13.65	Other specified diabetes mellitus with hyperglycemia
E13.69	Other specified diabetes mellitus with other specified complication
E10.620	Type 1 diabetes mellitus with diabetic dermatitis
E10.621	Type 1 diabetes mellitus with foot ulcer
E10.622	Type 1 diabetes mellitus with other skin ulcer
E10.628	Type 1 diabetes mellitus with other skin complications
E10.638	Type 1 diabetes mellitus with other oral complications
E10.649	Type 1 diabetes mellitus with hypoglycemia without coma
E11.8	Type 2 diabetes mellitus with unspecified complications
E13.8	Other specified diabetes mellitus with unspecified complications
E16.2	Hypoglycemia, unspecified
M10.9	Gout, unspecified
G44.209	Tension-type headache, unspecified, not intractable
G43.909	Migraine, unspecified, not intractable, without status migrainosus
G51.0	Bell's palsy
G56.00	Carpal tunnel syndrome, unspecified upper limb
G56.01	Carpal tunnel syndrome, right upper limb
G56.02	Carpal tunnel syndrome, left upper limb
G56.90	Unspecified mononeuropathy of unspecified upper limb
G56.91	Unspecified mononeuropathy of right upper limb
G56.92	Unspecified mononeuropathy of left upper limb
H10.30	Unspecified acute conjunctivitis, unspecified eye
H10.31	Unspecified acute conjunctivitis, right eye
H10.32	Unspecified acute conjunctivitis, left eye
H10.33	Unspecified acute conjunctivitis, bilateral
H10.021	Other mucopurulent conjunctivitis, right eye
H10.022	Other mucopurulent conjunctivitis, left eye
H10.023	Other mucopurulent conjunctivitis, bilateral
H10.029	Other mucopurulent conjunctivitis, unspecified eye
H10.411	Chronic giant papillary conjunctivitis, right eye

© 2023 Pomerantz A et al. *JAMA Network Open*.

H10.412	Chronic giant papillary conjunctivitis, left eye
H10.413	Chronic giant papillary conjunctivitis, bilateral
H10.419	Chronic giant papillary conjunctivitis, unspecified eye
H10.45	Other chronic allergic conjunctivitis
H10.9	Unspecified conjunctivitis
H11.001	Unspecified pterygium of right eye
H11.002	Unspecified pterygium of left eye
H11.003	Unspecified pterygium of eye, bilateral
H11.009	Unspecified pterygium of unspecified eye
H11.011	Amyloid pterygium of right eye
H11.012	Amyloid pterygium of left eye
H11.013	Amyloid pterygium of eye, bilateral
H11.019	Amyloid pterygium of unspecified eye
H00.011	Hordeolum externum right upper eyelid
H00.012	Hordeolum externum right lower eyelid
H00.013	Hordeolum externum right eye, unspecified eyelid
H00.014	Hordeolum externum left upper eyelid
H00.015	Hordeolum externum left lower eyelid
H00.016	Hordeolum externum left eye, unspecified eyelid
H00.019	Hordeolum externum unspecified eye, unspecified eyelid
H00.031	Abscess of right upper eyelid
H00.032	Abscess of right lower eyelid
H00.033	Abscess of eyelid right eye, unspecified eyelid
H00.034	Abscess of left upper eyelid
H00.035	Abscess of left lower eyelid
H00.036	Abscess of eyelid left eye, unspecified eyelid
H00.039	Abscess of eyelid unspecified eye, unspecified eyelid
H00.11	Chalazion right upper eyelid
H00.12	Chalazion right lower eyelid
H00.13	Chalazion right eye, unspecified eyelid

© 2023 Pomerantz A et al. *JAMA Network Open*.

H00.14	Chalazion left upper eyelid
H00.15	Chalazion left lower eyelid
H00.16	Chalazion left eye, unspecified eyelid
H00.19	Chalazion unspecified eye, unspecified eyelid
H57.10	Ocular pain, unspecified eye
H57.11	Ocular pain, right eye
H57.12	Ocular pain, left eye
H57.13	Ocular pain, bilateral
H60.00	Abscess of external ear, unspecified ear
H60.01	Abscess of right external ear
H60.02	Abscess of left external ear
H60.03	Abscess of external ear, bilateral
H60.10	Cellulitis of external ear, unspecified ear
H60.11	Cellulitis of right external ear
H60.12	Cellulitis of left external ear
H60.13	Cellulitis of external ear, bilateral
H60.311	Diffuse otitis externa, right ear
H60.312	Diffuse otitis externa, left ear
H60.313	Diffuse otitis externa, bilateral
H60.319	Diffuse otitis externa, unspecified ear
H60.321	Hemorrhagic otitis externa, right ear
H60.322	Hemorrhagic otitis externa, left ear
H60.323	Hemorrhagic otitis externa, bilateral
H60.329	Hemorrhagic otitis externa, unspecified ear
H60.391	Other infective otitis externa, right ear
H60.392	Other infective otitis externa, left ear
H60.393	Other infective otitis externa, bilateral
H60.399	Other infective otitis externa, unspecified ear
H61.20	Impacted cerumen, unspecified ear
H61.21	Impacted cerumen, right ear

© 2023 Pomerantz A et al. *JAMA Network Open*.

H61.22	Impacted cerumen, left ear
H61.23	Impacted cerumen, bilateral
H65.191	Other acute nonsuppurative otitis media, right ear
H65.192	Other acute nonsuppurative otitis media, left ear
H65.193	Other acute nonsuppurative otitis media, bilateral
H65.194	Other acute nonsuppurative otitis media, recurrent, right ear
H65.195	Other acute nonsuppurative otitis media, recurrent, left ear
H65.196	Other acute nonsuppurative otitis media, recurrent, bilateral
H65.197	Other acute nonsuppurative otitis media recurrent, unspecified ear
H65.199	Other acute nonsuppurative otitis media, unspecified ear
H65.00	Acute serous otitis media, unspecified ear
H65.01	Acute serous otitis media, right ear
H65.02	Acute serous otitis media, left ear
H65.03	Acute serous otitis media, bilateral
H65.04	Acute serous otitis media, recurrent, right ear
H65.05	Acute serous otitis media, recurrent, left ear
H65.06	Acute serous otitis media, recurrent, bilateral
H65.07	Acute serous otitis media, recurrent, unspecified ear
H65.20	Chronic serous otitis media, unspecified ear
H65.21	Chronic serous otitis media, right ear
H65.22	Chronic serous otitis media, left ear
H65.23	Chronic serous otitis media, bilateral
H65.90	Unspecified nonsuppurative otitis media, unspecified ear
H65.91	Unspecified nonsuppurative otitis media, right ear
H65.92	Unspecified nonsuppurative otitis media, left ear
H65.93	Unspecified nonsuppurative otitis media, bilateral
H66.001	Acute suppurative otitis media without spontaneous rupture of ear drum, right ear
H66.002	Acute suppurative otitis media without spontaneous rupture of ear drum, left ear
H66.003	Acute suppurative otitis media without spontaneous rupture of ear drum, bilateral
H66.004	Acute suppurative otitis media without spontaneous rupture of ear drum, recurrent, right ear

© 2023 Pomerantz A et al. *JAMA Network Open*.

H66.005	Acute suppurative otitis media without spontaneous rupture of ear drum, recurrent, left ear
H66.006	Acute suppurative otitis media without spontaneous rupture of ear drum, recurrent, bilateral
H66.007	Acute suppurative otitis media without spontaneous rupture of ear drum, recurrent, unspecified ear
H66.009	Acute suppurative otitis media without spontaneous rupture of ear drum, unspecified ear
H66.90	Otitis media, unspecified, unspecified ear
H66.91	Otitis media, unspecified, right ear
H66.92	Otitis media, unspecified, left ear
H66.93	Otitis media, unspecified, bilateral
H72.90	Unspecified perforation of tympanic membrane, unspecified ear
H72.91	Unspecified perforation of tympanic membrane, right ear
H72.92	Unspecified perforation of tympanic membrane, left ear
H72.93	Unspecified perforation of tympanic membrane, bilateral
H83.3X1	Noise effects on right inner ear
H83.3X2	Noise effects on left inner ear
H83.3X3	Noise effects on inner ear, bilateral
H83.3X9	Noise effects on inner ear, unspecified ear
H93.11	Tinnitus, right ear
H93.12	Tinnitus, left ear
H93.13	Tinnitus, bilateral
H93.19	Tinnitus, unspecified ear
H92.10	Otorrhea, unspecified ear
H92.11	Otorrhea, right ear
H92.12	Otorrhea, left ear
H92.13	Otorrhea, bilateral
H92.20	Otorrhagia, unspecified ear
H92.21	Otorrhagia, right ear
H92.22	Otorrhagia, left ear
H92.23	Otorrhagia, bilateral

© 2023 Pomerantz A et al. *JAMA Network Open*.

H92.01	Otalgia, right ear
H92.02	Otalgia, left ear
H92.03	Otalgia, bilateral
H92.09	Otalgia, unspecified ear
H93.8X1	Other specified disorders of right ear
H93.8X2	Other specified disorders of left ear
H93.8X3	Other specified disorders of ear, bilateral
H93.8X9	Other specified disorders of ear, unspecified ear
H94.80	Other specified disorders of ear in diseases classified elsewhere, unspecified ear
H94.81	Other specified disorders of right ear in diseases classified elsewhere
H94.82	Other specified disorders of left ear in diseases classified elsewhere
H94.83	Other specified disorders of ear in diseases classified elsewhere, bilateral
I10.	Essential (primary) hypertension
I50.9	Heart failure, unspecified
K64.9	Unspecified hemorrhoids
J00.	Acute nasopharyngitis [common cold]
J01.00	Acute maxillary sinusitis, unspecified
J01.01	Acute recurrent maxillary sinusitis
J01.90	Acute sinusitis, unspecified
J01.91	Acute recurrent sinusitis, unspecified
J02.8	Acute pharyngitis due to other specified organisms
J02.9	Acute pharyngitis, unspecified
J03.80	Acute tonsillitis due to other specified organisms
J03.81	Acute recurrent tonsillitis due to other specified organisms
J03.90	Acute tonsillitis, unspecified
J03.91	Acute recurrent tonsillitis, unspecified
J04.10	Acute tracheitis without obstruction
J06.9	Acute upper respiratory infection, unspecified
J20.8	Acute bronchitis due to other specified organisms
J20.9	Acute bronchitis, unspecified

© 2023 Pomerantz A et al. *JAMA Network Open*.

J31.0	Chronic rhinitis
J32.0	Chronic maxillary sinusitis
J32.9	Chronic sinusitis, unspecified
J30.1	Allergic rhinitis due to pollen
J30.0	Vasomotor rhinitis
J30.9	Allergic rhinitis, unspecified
J18.1	Lobar pneumonia, unspecified organism
J18.0	Bronchopneumonia, unspecified organism
J18.8	Other pneumonia, unspecified organism
J18.9	Pneumonia, unspecified organism
J10.1	Influenza due to other identified influenza virus with other respiratory manifestations
J11.1	Influenza due to unidentified influenza virus with other respiratory manifestations
J40.	Bronchitis, not specified as acute or chronic
J44.9	Chronic obstructive pulmonary disease, unspecified
J44.1	Chronic obstructive pulmonary disease with (acute) exacerbation
J42.	Unspecified chronic bronchitis
J43.9	Emphysema, unspecified
J43.0	Unilateral pulmonary emphysema [MacLeod's syndrome]
J43.1	Panlobular emphysema
J43.2	Centrilobular emphysema
J43.8	Other emphysema
J45.20	Mild intermittent asthma, uncomplicated
J45.30	Mild persistent asthma, uncomplicated
J45.40	Moderate persistent asthma, uncomplicated
J45.50	Severe persistent asthma, uncomplicated
J45.22	Mild intermittent asthma with status asthmaticus
J45.32	Mild persistent asthma with status asthmaticus
J45.42	Moderate persistent asthma with status asthmaticus
J45.52	Severe persistent asthma with status asthmaticus
J45.21	Mild intermittent asthma with (acute) exacerbation

© 2023 Pomerantz A et al. *JAMA Network Open*.

J45.31	Mild persistent asthma with (acute) exacerbation
J45.41	Moderate persistent asthma with (acute) exacerbation
J45.51	Severe persistent asthma with (acute) exacerbation
J45.990	Exercise induced bronchospasm
J45.991	Cough variant asthma
J45.909	Unspecified asthma, uncomplicated
J45.998	Other asthma
J45.902	Unspecified asthma with status asthmaticus
J45.901	Unspecified asthma with (acute) exacerbation
K04.4	Acute apical periodontitis of pulpal origin
K04.7	Periapical abscess without sinus
K08.8	Other specified disorders of teeth and supporting structures
M26.79	Other specified alveolar anomalies
K08.9	Disorder of teeth and supporting structures, unspecified
K12.2	Cellulitis and abscess of mouth
K12.0	Recurrent oral aphthae
K13.1	Cheek and lip biting
K13.4	Granuloma and granuloma-like lesions of oral mucosa
K13.6	Irritative hyperplasia of oral mucosa
K13.70	Unspecified lesions of oral mucosa
K13.79	Other lesions of oral mucosa
K21.9	Gastro-esophageal reflux disease without esophagitis
K40.90	Unilateral inguinal hernia, without obstruction or gangrene, not specified as recurrent
K52.89	Other specified noninfective gastroenteritis and colitis
K52.9	Noninfective gastroenteritis and colitis, unspecified
K58.0	Irritable bowel syndrome with diarrhea
K58.9	Irritable bowel syndrome without diarrhea
K60.0	Acute anal fissure
K60.1	Chronic anal fissure
K60.2	Anal fissure, unspecified

© 2023 Pomerantz A et al. *JAMA Network Open*.

N10.	Acute tubulo-interstitial nephritis
N11.9	Chronic tubulo-interstitial nephritis, unspecified
N12.	Tubulo-interstitial nephritis, not specified as acute or chronic
N13.6	Pyonephrosis
N30.00	Acute cystitis without hematuria
N30.01	Acute cystitis with hematuria
N30.90	Cystitis, unspecified without hematuria
N30.91	Cystitis, unspecified with hematuria
N34.1	Nonspecific urethritis
N34.2	Other urethritis
N39.0	Urinary tract infection, site not specified
N45.1	Epididymitis
N45.2	Orchitis
N45.3	Epididymo-orchitis
N47.6	Balanoposthitis
N48.1	Balanitis
N50.9	Disorder of male genital organs, unspecified
R10.2	Pelvic and perineal pain
N64.4	Mastodynia
N63.	Unspecified lump in breast
N73.5	Female pelvic peritonitis, unspecified
N73.9	Female pelvic inflammatory disease, unspecified
N72.	Inflammatory disease of cervix uteri
N76.0	Acute vaginitis
N76.1	Subacute and chronic vaginitis
N76.2	Acute vulvitis
N76.3	Subacute and chronic vulvitis
N83.20	Unspecified ovarian cysts
N83.29	Other ovarian cysts
N89.8	Other specified noninflammatory disorders of vagina

© 2023 Pomerantz A et al. *JAMA Network Open*.

N94.4	Primary dysmenorrhea
N94.5	Secondary dysmenorrhea
N94.6	Dysmenorrhea, unspecified
N94.89	Other specified conditions associated with female genital organs and menstrual cycle
N92.0	Excessive and frequent menstruation with regular cycle
N92.5	Other specified irregular menstruation
N92.6	Irregular menstruation, unspecified
N89.7	Hematocolpos
N93.8	Other specified abnormal uterine and vaginal bleeding
N93.9	Abnormal uterine and vaginal bleeding, unspecified
O21.0	Mild hyperemesis gravidarum
O25.11	Malnutrition in pregnancy, first trimester
O25.12	Malnutrition in pregnancy, second trimester
O25.13	Malnutrition in pregnancy, third trimester
O99.281	Endocrine, nutritional and metabolic diseases complicating pregnancy, first trimester
O99.282	Endocrine, nutritional and metabolic diseases complicating pregnancy, second trimester
O99.283	Endocrine, nutritional and metabolic diseases complicating pregnancy, third trimester
O99.511	Diseases of the respiratory system complicating pregnancy, first trimester
O99.512	Diseases of the respiratory system complicating pregnancy, second trimester
O99.513	Diseases of the respiratory system complicating pregnancy, third trimester
O99.611	Diseases of the digestive system complicating pregnancy, first trimester
O99.612	Diseases of the digestive system complicating pregnancy, second trimester
O99.613	Diseases of the digestive system complicating pregnancy, third trimester
O99.711	Diseases of the skin and subcutaneous tissue complicating pregnancy, first trimester
O99.712	Diseases of the skin and subcutaneous tissue complicating pregnancy, second trimester
O99.713	Diseases of the skin and subcutaneous tissue complicating pregnancy, third trimester
O9A.111	Malignant neoplasm complicating pregnancy, first trimester
O9A.112	Malignant neoplasm complicating pregnancy, second trimester
O9A.113	Malignant neoplasm complicating pregnancy, third trimester
O9A.211	Injury, poisoning and certain other consequences of external causes complicating pregnancy, first trimester

© 2023 Pomerantz A et al. *JAMA Network Open*.

O9A.212	Injury, poisoning and certain other consequences of external causes complicating pregnancy, second trimester
O9A.213	Injury, poisoning and certain other consequences of external causes complicating pregnancy, third trimester
L02.92	Furuncle, unspecified
L02.93	Carbuncle, unspecified
L02.511	Cutaneous abscess of right hand
L02.512	Cutaneous abscess of left hand
L02.519	Cutaneous abscess of unspecified hand
L03.011	Cellulitis of right finger
L03.012	Cellulitis of left finger
L03.019	Cellulitis of unspecified finger
L03.021	Acute lymphangitis of right finger
L03.022	Acute lymphangitis of left finger
L03.029	Acute lymphangitis of unspecified finger
L02.611	Cutaneous abscess of right foot
L02.612	Cutaneous abscess of left foot
L02.619	Cutaneous abscess of unspecified foot
L03.031	Cellulitis of right toe
L03.032	Cellulitis of left toe
L03.039	Cellulitis of unspecified toe
L03.041	Acute lymphangitis of right toe
L03.042	Acute lymphangitis of left toe
L03.049	Acute lymphangitis of unspecified toe
L02.01	Cutaneous abscess of face
L03.211	Cellulitis of face
L03.212	Acute lymphangitis of face
L02.211	Cutaneous abscess of abdominal wall
L02.212	Cutaneous abscess of back [any part, except buttock]
L02.213	Cutaneous abscess of chest wall
L02.214	Cutaneous abscess of groin

© 2023 Pomerantz A et al. *JAMA Network Open*.

L02.215	Cutaneous abscess of perineum
L02.216	Cutaneous abscess of umbilicus
L02.219	Cutaneous abscess of trunk, unspecified
L03.311	Cellulitis of abdominal wall
L03.312	Cellulitis of back [any part except buttock]
L03.313	Cellulitis of chest wall
L03.314	Cellulitis of groin
L03.315	Cellulitis of perineum
L03.316	Cellulitis of umbilicus
L03.319	Cellulitis of trunk, unspecified
L03.321	Acute lymphangitis of abdominal wall
L03.322	Acute lymphangitis of back [any part except buttock]
L03.323	Acute lymphangitis of chest wall
L03.324	Acute lymphangitis of groin
L03.325	Acute lymphangitis of perineum
L03.326	Acute lymphangitis of umbilicus
L03.329	Acute lymphangitis of trunk, unspecified
L02.411	Cutaneous abscess of right axilla
L02.412	Cutaneous abscess of left axilla
L02.413	Cutaneous abscess of right upper limb
L02.414	Cutaneous abscess of left upper limb
L02.419	Cutaneous abscess of limb, unspecified
L03.111	Cellulitis of right axilla
L03.112	Cellulitis of left axilla
L03.113	Cellulitis of right upper limb
L03.114	Cellulitis of left upper limb
L03.119	Cellulitis of unspecified part of limb
L03.121	Acute lymphangitis of right axilla
L03.122	Acute lymphangitis of left axilla
L03.123	Acute lymphangitis of right upper limb

© 2023 Pomerantz A et al. *JAMA Network Open*.

L03.124	Acute lymphangitis of left upper limb
L03.129	Acute lymphangitis of unspecified part of limb
L02.31	Cutaneous abscess of buttock
L03.317	Cellulitis of buttock
L03.327	Acute lymphangitis of buttock
L02.415	Cutaneous abscess of right lower limb
L02.416	Cutaneous abscess of left lower limb
L03.115	Cellulitis of right lower limb
L03.116	Cellulitis of left lower limb
L03.125	Acute lymphangitis of right lower limb
L03.126	Acute lymphangitis of left lower limb
L02.811	Cutaneous abscess of head [any part, except face]
L02.818	Cutaneous abscess of other sites
L03.811	Cellulitis of head [any part, except face]
L03.818	Cellulitis of other sites
L03.891	Acute lymphangitis of head [any part, except face]
L03.898	Acute lymphangitis of other sites
L02.91	Cutaneous abscess, unspecified
L03.90	Cellulitis, unspecified
L03.91	Acute lymphangitis, unspecified
L98.3	Eosinophilic cellulitis [Wells]
L01.00	Impetigo, unspecified
L01.01	Non-bullous impetigo
L01.02	Bockhart's impetigo
L01.03	Bullous impetigo
L01.09	Other impetigo
L01.1	Impetiginization of other dermatoses
L05.01	Pilonidal cyst with abscess
L05.02	Pilonidal sinus with abscess
L05.91	Pilonidal cyst without abscess

© 2023 Pomerantz A et al. *JAMA Network Open*.

L05.92	Pilonidal sinus without abscess
L08.9	Local infection of the skin and subcutaneous tissue, unspecified
L21.9	Seborrheic dermatitis, unspecified
L22.	Diaper dermatitis
L20.0	Besnier's prurigo
L20.81	Atopic neurodermatitis
L20.82	Flexural eczema
L20.84	Intrinsic (allergic) eczema
L20.89	Other atopic dermatitis
L20.9	Atopic dermatitis, unspecified
L23.7	Allergic contact dermatitis due to plants, except food
L24.7	Irritant contact dermatitis due to plants, except food
L25.5	Unspecified contact dermatitis due to plants, except food
L55.0	Sunburn of first degree
L55.9	Sunburn, unspecified
L23.9	Allergic contact dermatitis, unspecified cause
L24.9	Irritant contact dermatitis, unspecified cause
L25.9	Unspecified contact dermatitis, unspecified cause
L30.0	Nummular dermatitis
L30.2	Cutaneous autosensitization
L30.8	Other specified dermatitis
L30.9	Dermatitis, unspecified
L27.0	Generalized skin eruption due to drugs and medicaments taken internally
L27.1	Localized skin eruption due to drugs and medicaments taken internally
L27.2	Dermatitis due to ingested food
L42.	Pityriasis rosea
L29.9	Pruritus, unspecified
L60.0	Ingrowing nail
L63.2	Ophiasis
L63.8	Other alopecia areata

© 2023 Pomerantz A et al. *JAMA Network Open*.

L63.9	Alopecia areata, unspecified
L66.3	Perifolliculitis capitis abscedens
L73.1	Pseudofolliculitis barbae
L73.8	Other specified follicular disorders
L74.0	Miliaria rubra
L74.1	Miliaria crystallina
L74.2	Miliaria profunda
L74.3	Miliaria, unspecified
L74.8	Other eccrine sweat disorders
L75.0	Bromhidrosis
L75.1	Chromhidrosis
L75.8	Other apocrine sweat disorders
L70.0	Acne vulgaris
L70.1	Acne conglobata
L70.3	Acne tropica
L70.4	Infantile acne
L70.5	Acne excoriee des jeunes filles
L70.8	Other acne
L70.9	Acne, unspecified
L73.0	Acne keloid
L72.0	Epidermal cyst
L72.2	Steatocystoma multiplex
L72.3	Sebaceous cyst
L72.8	Other follicular cysts of the skin and subcutaneous tissue
L72.9	Follicular cyst of the skin and subcutaneous tissue, unspecified
L50.9	Urticaria, unspecified
M12.9	Arthropathy, unspecified
M22.90	Unspecified disorder of patella, unspecified knee
M22.91	Unspecified disorder of patella, right knee
M22.92	Unspecified disorder of patella, left knee

© 2023 Pomerantz A et al. *JAMA Network Open*.

M23.90	Unspecified internal derangement of unspecified knee
M23.91	Unspecified internal derangement of right knee
M23.92	Unspecified internal derangement of left knee
M25.461	Effusion, right knee
M25.462	Effusion, left knee
M25.469	Effusion, unspecified knee
M25.511	Pain in right shoulder
M25.512	Pain in left shoulder
M25.519	Pain in unspecified shoulder
M25.521	Pain in right elbow
M25.522	Pain in left elbow
M25.529	Pain in unspecified elbow
M25.531	Pain in right wrist
M25.532	Pain in left wrist
M25.539	Pain in unspecified wrist
M25.561	Pain in right knee
M25.562	Pain in left knee
M25.569	Pain in unspecified knee
M25.571	Pain in right ankle and joints of right foot
M25.572	Pain in left ankle and joints of left foot
M25.579	Pain in unspecified ankle and joints of unspecified foot
M25.50	Pain in unspecified joint
M54.2	Cervicalgia
M54.5	Low back pain
M54.14	Radiculopathy, thoracic region
M54.15	Radiculopathy, thoracolumbar region
M54.16	Radiculopathy, lumbar region
M54.17	Radiculopathy, lumbosacral region
M54.89	Other dorsalgia
M54.9	Dorsalgia, unspecified

© 2023 Pomerantz A et al. *JAMA Network Open*.

M54.03	Panniculitis affecting regions of neck and back, cervicothoracic region
M54.04	Panniculitis affecting regions of neck and back, thoracic region
M54.05	Panniculitis affecting regions of neck and back, thoracolumbar region
M54.06	Panniculitis affecting regions of neck and back, lumbar region
M54.07	Panniculitis affecting regions of neck and back, lumbosacral region
M54.08	Panniculitis affecting regions of neck and back, sacral and sacrococcygeal region
M54.09	Panniculitis affecting regions, neck and back, multiple sites in spine
M62.830	Muscle spasm of back
M25.751	Osteophyte, right hip
M25.752	Osteophyte, left hip
M25.759	Osteophyte, unspecified hip
M70.60	Trochanteric bursitis, unspecified hip
M70.61	Trochanteric bursitis, right hip
M70.62	Trochanteric bursitis, left hip
M70.70	Other bursitis of hip, unspecified hip
M70.71	Other bursitis of hip, right hip
M70.72	Other bursitis of hip, left hip
M76.00	Gluteal tendinitis, unspecified hip
M76.01	Gluteal tendinitis, right hip
M76.02	Gluteal tendinitis, left hip
M76.10	Psoas tendinitis, unspecified hip
M76.11	Psoas tendinitis, right hip
M76.12	Psoas tendinitis, left hip
M76.20	Iliac crest spur, unspecified hip
M76.21	Iliac crest spur, right hip
M76.22	Iliac crest spur, left hip
M76.30	Iliotibial band syndrome, unspecified leg
M76.31	Iliotibial band syndrome, right leg
M76.32	Iliotibial band syndrome, left leg
M76.50	Patellar tendinitis, unspecified knee

© 2023 Pomerantz A et al. *JAMA Network Open*.

M76.51	Patellar tendinitis, right knee
M76.52	Patellar tendinitis, left knee
M76.70	Peroneal tendinitis, unspecified leg
M76.71	Peroneal tendinitis, right leg
M76.72	Peroneal tendinitis, left leg
M77.50	Other enthesopathy of unspecified foot
M77.51	Other enthesopathy of right foot
M77.52	Other enthesopathy of left foot
M77.9	Enthesopathy, unspecified
M25.70	Osteophyte, unspecified joint
M65.831	Other synovitis and tenosynovitis, right forearm
M65.832	Other synovitis and tenosynovitis, left forearm
M65.839	Other synovitis and tenosynovitis, unspecified forearm
M65.841	Other synovitis and tenosynovitis, right hand
M65.842	Other synovitis and tenosynovitis, left hand
M65.849	Other synovitis and tenosynovitis, unspecified hand
M65.10	Other infective (teno)synovitis, unspecified site
M65.111	Other infective (teno)synovitis, right shoulder
M65.112	Other infective (teno)synovitis, left shoulder
M65.119	Other infective (teno)synovitis, unspecified shoulder
M65.121	Other infective (teno)synovitis, right elbow
M65.122	Other infective (teno)synovitis, left elbow
M65.129	Other infective (teno)synovitis, unspecified elbow
M65.131	Other infective (teno)synovitis, right wrist
M65.132	Other infective (teno)synovitis, left wrist
M65.139	Other infective (teno)synovitis, unspecified wrist
M65.141	Other infective (teno)synovitis, right hand
M65.142	Other infective (teno)synovitis, left hand
M65.149	Other infective (teno)synovitis, unspecified hand
M65.151	Other infective (teno)synovitis, right hip

© 2023 Pomerantz A et al. *JAMA Network Open*.

M65.152	Other infective (teno)synovitis, left hip
M65.159	Other infective (teno)synovitis, unspecified hip
M65.161	Other infective (teno)synovitis, right knee
M65.162	Other infective (teno)synovitis, left knee
M65.169	Other infective (teno)synovitis, unspecified knee
M65.171	Other infective (teno)synovitis, right ankle and foot
M65.172	Other infective (teno)synovitis, left ankle and foot
M65.179	Other infective (teno)synovitis, unspecified ankle and foot
M65.18	Other infective (teno)synovitis, other site
M65.19	Other infective (teno)synovitis, multiple sites
M65.80	Other synovitis and tenosynovitis, unspecified site
M65.811	Other synovitis and tenosynovitis, right shoulder
M65.812	Other synovitis and tenosynovitis, left shoulder
M65.819	Other synovitis and tenosynovitis, unspecified shoulder
M65.821	Other synovitis and tenosynovitis, right upper arm
M65.822	Other synovitis and tenosynovitis, left upper arm
M65.829	Other synovitis and tenosynovitis, unspecified upper arm
M65.851	Other synovitis and tenosynovitis, right thigh
M65.852	Other synovitis and tenosynovitis, left thigh
M65.859	Other synovitis and tenosynovitis, unspecified thigh
M65.861	Other synovitis and tenosynovitis, right lower leg
M65.862	Other synovitis and tenosynovitis, left lower leg
M65.869	Other synovitis and tenosynovitis, unspecified lower leg
M65.88	Other synovitis and tenosynovitis, other site
M65.89	Other synovitis and tenosynovitis, multiple sites
M67.30	Transient synovitis, unspecified site
M67.311	Transient synovitis, right shoulder
M67.312	Transient synovitis, left shoulder
M67.319	Transient synovitis, unspecified shoulder
M67.321	Transient synovitis, right elbow

© 2023 Pomerantz A et al. *JAMA Network Open*.

M67.322	Transient synovitis, left elbow
M67.329	Transient synovitis, unspecified elbow
M67.331	Transient synovitis, right wrist
M67.332	Transient synovitis, left wrist
M67.339	Transient synovitis, unspecified wrist
M67.341	Transient synovitis, right hand
M67.342	Transient synovitis, left hand
M67.349	Transient synovitis, unspecified hand
M67.351	Transient synovitis, right hip
M67.352	Transient synovitis, left hip
M67.359	Transient synovitis, unspecified hip
M67.361	Transient synovitis, right knee
M67.362	Transient synovitis, left knee
M67.369	Transient synovitis, unspecified knee
M67.371	Transient synovitis, right ankle and foot
M67.372	Transient synovitis, left ankle and foot
M67.379	Transient synovitis, unspecified ankle and foot
M67.38	Transient synovitis, other site
M67.39	Transient synovitis, multiple sites
M62.40	Contracture of muscle, unspecified site
M62.411	Contracture of muscle, right shoulder
M62.412	Contracture of muscle, left shoulder
M62.419	Contracture of muscle, unspecified shoulder
M62.421	Contracture of muscle, right upper arm
M62.422	Contracture of muscle, left upper arm
M62.429	Contracture of muscle, unspecified upper arm
M62.431	Contracture of muscle, right forearm
M62.432	Contracture of muscle, left forearm
M62.439	Contracture of muscle, unspecified forearm
M62.441	Contracture of muscle, right hand

© 2023 Pomerantz A et al. *JAMA Network Open*.

M62.442	Contracture of muscle, left hand
M62.449	Contracture of muscle, unspecified hand
M62.451	Contracture of muscle, right thigh
M62.452	Contracture of muscle, left thigh
M62.459	Contracture of muscle, unspecified thigh
M62.461	Contracture of muscle, right lower leg
M62.462	Contracture of muscle, left lower leg
M62.469	Contracture of muscle, unspecified lower leg
M62.471	Contracture of muscle, right ankle and foot
M62.472	Contracture of muscle, left ankle and foot
M62.479	Contracture of muscle, unspecified ankle and foot
M62.48	Contracture of muscle, other site
M62.49	Contracture of muscle, multiple sites
M62.831	Muscle spasm of calf
M62.838	Other muscle spasm
M60.80	Other myositis, unspecified site
M60.811	Other myositis, right shoulder
M60.812	Other myositis, left shoulder
M60.819	Other myositis, unspecified shoulder
M60.821	Other myositis, right upper arm
M60.822	Other myositis, left upper arm
M60.829	Other myositis, unspecified upper arm
M60.831	Other myositis, right forearm
M60.832	Other myositis, left forearm
M60.839	Other myositis, unspecified forearm
M60.841	Other myositis, right hand
M60.842	Other myositis, left hand
M60.849	Other myositis, unspecified hand
M60.851	Other myositis, right thigh
M60.852	Other myositis, left thigh

© 2023 Pomerantz A et al. *JAMA Network Open*.

M60.859	Other myositis, unspecified thigh
M60.861	Other myositis, right lower leg
M60.862	Other myositis, left lower leg
M60.869	Other myositis, unspecified lower leg
M60.871	Other myositis, right ankle and foot
M60.872	Other myositis, left ankle and foot
M60.879	Other myositis, unspecified ankle and foot
M60.88	Other myositis, other site
M60.89	Other myositis, multiple sites
M60.9	Myositis, unspecified
M79.1	Myalgia
M79.7	Fibromyalgia
M79.601	Pain in right arm
M79.602	Pain in left arm
M79.603	Pain in arm, unspecified
M79.604	Pain in right leg
M79.605	Pain in left leg
M79.606	Pain in leg, unspecified
M79.609	Pain in unspecified limb
M79.621	Pain in right upper arm
M79.622	Pain in left upper arm
M79.629	Pain in unspecified upper arm
M79.631	Pain in right forearm
M79.632	Pain in left forearm
M79.639	Pain in unspecified forearm
M79.641	Pain in right hand
M79.642	Pain in left hand
M79.643	Pain in unspecified hand
M79.644	Pain in right finger(s)
M79.645	Pain in left finger(s)

© 2023 Pomerantz A et al. *JAMA Network Open*.

M79.646	Pain in unspecified finger(s)
M79.651	Pain in right thigh
M79.652	Pain in left thigh
M79.659	Pain in unspecified thigh
M79.661	Pain in right lower leg
M79.662	Pain in left lower leg
M79.669	Pain in unspecified lower leg
M79.671	Pain in right foot
M79.672	Pain in left foot
M79.673	Pain in unspecified foot
M79.674	Pain in right toe(s)
M79.675	Pain in left toe(s)
M79.676	Pain in unspecified toe(s)
M79.89	Other specified soft tissue disorders
M94.0	Chondrocostal junction syndrome [Tietze]
R42.	Dizziness and giddiness
G93.3	Postviral fatigue syndrome
R53.0	Neoplastic (malignant) related fatigue
R53.1	Weakness
R53.81	Other malaise
R53.83	Other fatigue
R21.	Rash and other nonspecific skin eruption
R22.0	Localized swelling, mass and lump, head
R22.1	Localized swelling, mass and lump, neck
R22.30	Localized swelling, mass and lump, unspecified upper limb
R22.31	Localized swelling, mass and lump, right upper limb
R22.32	Localized swelling, mass and lump, left upper limb
R22.33	Localized swelling, mass and lump, upper limb, bilateral
R22.40	Localized swelling, mass and lump, unspecified lower limb
R22.41	Localized swelling, mass and lump, right lower limb

© 2023 Pomerantz A et al. *JAMA Network Open*.

R22.42	Localized swelling, mass and lump, left lower limb
R22.43	Localized swelling, mass and lump, lower limb, bilateral
R22.9	Localized swelling, mass and lump, unspecified
R23.3	Spontaneous ecchymoses
R23.4	Changes in skin texture
G44.1	Vascular headache, not elsewhere classified
R51.	Headache
R90.0	Intracranial space-occupying lesion found on diagnostic imaging of central nervous system
R04.0	Epistaxis
R59.0	Localized enlarged lymph nodes
R59.1	Generalized enlarged lymph nodes
R59.9	Enlarged lymph nodes, unspecified
R05.	Cough
R11.2	Nausea with vomiting, unspecified
R11.0	Nausea
R11.10	Vomiting, unspecified
R11.11	Vomiting without nausea
R11.12	Projectile vomiting
R14.0	Abdominal distension (gaseous)
R14.1	Gas pain
R14.2	Eructation
R14.3	Flatulence
R19.7	Diarrhea, unspecified
R19.4	Change in bowel habit
R30.0	Dysuria
R30.9	Painful micturition, unspecified
R35.0	Frequency of micturition
R35.8	Other polyuria
R35.1	Nocturia
R36.0	Urethral discharge without blood

© 2023 Pomerantz A et al. *JAMA Network Open*.

R36.9	Urethral discharge, unspecified
R10.0	Acute abdomen
R10.9	Unspecified abdominal pain
R10.11	Right upper quadrant pain
R10.12	Left upper quadrant pain
R10.31	Right lower quadrant pain
R10.32	Left lower quadrant pain
R10.13	Epigastric pain
R10.84	Generalized abdominal pain
R10.10	Upper abdominal pain, unspecified
R10.30	Lower abdominal pain, unspecified
R16.0	Hepatomegaly, not elsewhere classified
R19.00	Intra-abdominal and pelvic swelling, mass and lump, unspecified site
Z33.1	Pregnant state, incidental
Z76.0	Encounter for issue of repeat prescription

© 2023 Pomerantz A et al. *JAMA Network Open*.

eTable 2. Common Reasons for ED Visits Algorithmically Identified as Nonemergent

ICD-10 Code	Description	N (%)
J069	Acute upper respiratory infection, unspecified	798,141 (12.0)
R05	Cough	500,307 (7.5)
J029	Acute pharyngitis, unspecified	290,588 (4.4)
R109	Unspecified abdominal pain	242,177 (3.6)
J020	Streptococcal pharyngitis	235,336 (3.5)
R1110	Vomiting, unspecified	188,235 (2.8)
R51	Headache	158,676 (2.4)
R112	Nausea with vomiting, unspecified	156,487 (2.3)
H6691	Otitis media, unspecified, right ear	142,603 (2.1)
R21	Rash and other nonspecific skin eruption	133,569 (2.0)
	Total	2,846,119 (42.7)

© 2023 Pomerantz A et al. *JAMA Network Open*.

eTable 3. Algorithmically Identified Nonemergent ED Visits—Reimbursement Simulation

Billing Level	Average Reimbursement per Visit in US Dollars (Unadjusted)	Total Number of ED Visits	Number of ED Visits Identified as Nonemergent	Total Reimbursement in US Dollars	Total Decrease in Reimbursement from Reduction in US Dollars	Total Reimbursement after Reduction in US Dollars	Adjusted Average Reimbursement Per ED Visit in US Dollars	Relative Difference in Reimbursement in %
**Overall**								
2	28.56	673,490	271,156	19,234,874	3,939,897	15,294,978	22.71	-20.48
3	48.39	4,900,897	2,351,447	237,154,406	80,795,719	156,358,687	31.9	-34.08
4	83.59	2,896,999	1,415,366	242,160,146	98,452,859	143,707,287	49.61	-40.65
Total	58.87	8,471,386	4,037,969	498,549,427	183,188,475	315,360,952	37.23	-36.76
**Non-Hispanic White**								
2	28.56	343,060	129,455	9,797,794	1,880,981	7,916,812	23.08	-19.19
3	48.39	2,359,504	1,073,406	114,176,399	36,882,230	77,294,168	32.76	-32.30
4	83.59	1,421,833	665,766	118,851,020	46,310,683	72,540,338	51.02	-38.96
Total	58.87	4,124,397	1,868,627	242,825,213	85,073,894	157,751,318	38.25	-35.03
**Non-Hispanic Black**								
2	28.56	254,228	109,784	7,260,752	1,595,162	5,665,590	22.29	-21.95
3	48.39	1,972,587	996,638	95,453,485	34,244,482	61,209,003	31.03	-35.88
4	83.59	1,122,806	579,097	93,855,354	40,281,987	53,573,366	47.71	-42.92
Total	58.87	3,349,621	1,685,519	196,569,590	76,121,631	120,447,960	35.96	-38.92
**Hispanic**								
2	28.56	50,243	21,205	1,434,940	308,109	1,126,831	22.43	-21.46
3	48.39	366,838	183,464	17,751,291	6,303,823	11,447,468	31.21	-35.50
4	83.59	237,222	115,614	19,829,387	8,042,110	11,787,277	49.69	-40.56
Total	58.87	654,303	320,283	39,015,618	14,654,042	24,361,576	37.23	-36.76

© 2023 Pomerantz A et al. *JAMA Network Open*.
