# Supplementary material for: Racial and Ethnic Differences in Insurer Classification of Nonemergent Pediatric Emergency Department Visits
Source: JAMA Netw Open. 2023 May 4;6(5):e2311752. doi: 10.1001/jamanetworkopen.2023.11752 (PMC10160869; doi:10.1001/jamanetworkopen.2023.11752)
Supplement: Supplement 2. — Data Sharing Statement [file jamanetwopen-e2311752-s002.pdf]

Data Sharing Statement

Pomerantz. Racial and Ethnic Differences in Insurer Classification of Nonemergent Pediatric Emergency Department Visits. *JAMA Netw Open*. Published May 04, 2023.
doi:10.1001/jamanetworkopen.2023.11752

Data
Data available: No

Additional Information
Explanation for why data not available: This study uses information from a proprietary dataset and we would not be able to make this information available under the terms of use.
